# Development and Validation of a Semi-Automated Surveillance Algorithm for Cardiac Device Infections: Insights from the VA CART program

**DOI:** 10.1038/s41598-020-62083-y

**Published:** 2020-03-24

**Authors:** Archana Asundi, Maggie Stanislawski, Payal Mehta, Hillary J. Mull, Marin L. Schweizer, Anna E. Barón, P. Michael Ho, Kalpana Gupta, Westyn Branch-Elliman

**Affiliations:** 1 Division of Infectious Diseases, Boston Medical Center, Boston, Massachusetts USA; 2 Seattle-Denver Center of Innovation for Veteran-Centered and Value-Driven Care, Seattle, Washington and Aurora, Colorado USA; 3 Division of Biomedical Informatics and Personalized Medicine, University of Colorado School of Medicine, Aurora, Colorado USA; 4 Cardiology Section, Rocky Mountain Regional VA Medical Center, Aurora, Colorado USA; 5 Department of Medicine, Division of Infectious Diseases, Boston VA Healthcare System, West Roxbury, Massachusetts USA; 6 Center for Healthcare Organization and Implementation Research (CHOIR), Boston VA Healthcare System, Boston, Massachusetts USA; 7 Department of Surgery, Boston University School of Medicine, Boston, Massachusetts USA; 8 Center for Access and Delivery Research and Evaluation, Iowa City VA Health Care System, Iowa City, Iowa USA; 9 Department of Biostatistics & Informatics, Colorado School of Public Health, University of Colorado Anschutz Medical Campus, Aurora, Colorado USA; 10 Department of Medicine, Division of Cardiology, University of Colorado School of Medicine, Aurora, Colorado USA; 11 Boston University School of Medicine, Boston, Massachusetts USA; 12 Harvard Medical School, Boston, Massachusetts USA

**Keywords:** Cardiac device therapy, Epidemiology

## Abstract

Procedure-related cardiac electronic implantable device (CIED) infections have high morbidity and mortality, highlighting the urgent need for infection prevention efforts to include electrophysiology procedures. We developed and validated a semi-automated algorithm based on structured electronic health records data to reliably identify CIED infections. A sample of CIED procedures entered into the Veterans’ Health Administration Clinical Assessment Reporting and Tracking program from FY 2008–2015 was reviewed for the presence of CIED infection. This sample was then randomly divided into training (2/3) validation sets (1/3). The training set was used to develop a detection algorithm containing structured variables mapped from the clinical pathways of CIED infection. Performance of this algorithm was evaluated using the validation set. 2,107 unique CIED procedures from a cohort of 5,753 underwent manual review; 97 CIED infections (4.6%) were identified. Variables strongly associated with true infections included presence of a microbiology order, billing codes for surgical site infections and post-procedural antibiotic prescriptions. The combined algorithm to detect infection demonstrated high c-statistic (0.95; 95% confidence interval: 0.92–0.98), sensitivity (87.9%) and specificity (90.3%) in the validation data. Structured variables derived from clinical pathways can guide development of a semi-automated detection tool to surveil for CIED infection.

## Introduction

Cardiovascular electronic implantable devices (CIEDs), such as pacemakers and implantable automated defibrillators, are increasing as the population ages^1,2^. Procedure-related CIED infections are associated with high morbidity, mortality, and medical costs. Mortality rates for deep infections involving leads implanted into the heart approach 20% and infections are estimated to cost greater than $50,000^3–5^. CIED infections are increasingly a concern for cardiologists, infection control departments, and patient safety programs due to a trend toward an increasing rate of procedure-related infections^1^. The increased rate of CIED infections is compounded by the overall increasing rates of CIED placements^6^. Given this trend, developing and implementing effective infection prevention programs in the electrophysiology suite is crucial for minimizing morbidity and mortality among CIED implantation recipients.

**Figure 1:**
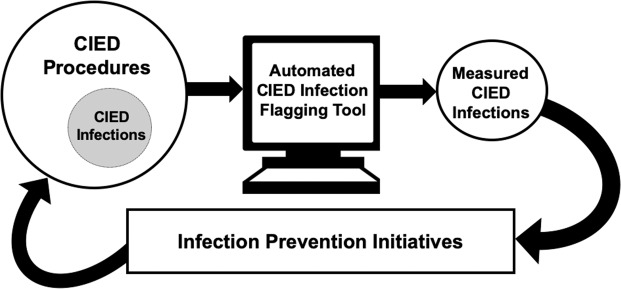
Automated electronic surveillance for CIED infections as a part of infection prevention and control efforts.

Surveillance with audit and feedback is a cornerstone of, and critical first step in, infection prevention (Fig. 1)^7^. Surveillance programs measure infections when they occur. This information can then be used to identify operational and patient-centered targets for prevention initiatives after infections and clusters are found. Surveillance programs can improve outcomes in several ways. They can be used to measure the effectiveness of novel infection prevention measures in real-time^8^ and can also provide feedback to providers to encourage uptake of evidence-based prevention practices^9^. Surveillance systems can also improve care through the observer, or Hawthorne, effect^10^. The Surgical Care Improvement Project (SCIP) infection metric bundle is an example of a surveillance and reporting system that lead to major improvements in surgical care and a reduction in post-operative infections^11^. However, due to the nature of the electrophysiology laboratory as a procedural area that straddles inpatient and outpatient settings, it is not encompassed by surgical site infection (SSI) quality improvement initiatives^12^.

Given limited resources, and the increasing dissemination of electronic health records (EHRs), a promising strategy for expanding surveillance to uncovered procedural areas is the development of surveillance tools that leverage clinical data warehouses to measure and track infections. These tools could be used as stand-alone systems or could be used in a semi-automated method to augment and triage the manual review process toward cases at highest probability of having an adverse event^7,13^. Thus, we sought to develop and validate a surveillance tool for CIED infection surveillance using structured data elements from the Veterans Health Administration (VA)’s Corporate Data Warehouse (CDW) in order to triage cases for manual review.

## Methods

### Study overview

This study used structured data elements along the diagnostic and treatment pathway of CIED infection management from the VA EHR captured in the CDW to develop an algorithm to detect infections. We used a limited definition of structured data focusing on diagnostic testing orders, laboratory results, pharmacy and billing data that is structured and standardized across all EHRs in order to ensure broad potential applicability of the detection algorithm. A sample of cardiac procedures collected as part of the VA Clinical Assessment Reporting and Tracking (CART) quality program underwent manual review for the presence of infection; these cases were used in the development and validation of the semi-automated tool.

### Ethical considerations

Given the retrospective nature of the study, waiver of consent was obtained. The Veterans Health Administration Boston Healthcare System Institutional Review Board and the University of Colorado Denver Anschutz Medical Campus Multiple Institution Review Board (covering the University of Colorado Denver and its affiliates: Children’s Hospital Colorado, Denver Health and Hospital Authority, University of Colorado Hospital, and the VA Eastern Colorado Health Care System) approved this study and waived the need for informed consent as part of study approval prior to data collection and analysis. The study was also approved by the VA CART-Electrophysiology (EP) program. All methods were carried out in accordance with relevant guidelines and regulations.

### Cohort development

Our cohort was a subset of CIED procedures captured by the CART program. CART is a national quality initiative integrated into the VA electronic medical record, which collects data about clinical outcomes, co-morbidities, and procedural details, but does not include measurement of infections. CART reporting is mandatory for all cardiac catheterization procedures and optional for EP procedures, including device implantations and revisions. Approximately 20% of EP device procedures performed across the national VA healthcare system are captured by CART-EP. Multiple clinical and procedural variables are captured and combined with other data from the VA CDW to create a single national data repository^14,15^.

CIED procedures, including implantations and revisions of permanent pacemakers, implantable cardioverter defibrillators, biventricular pacemaker-implantable cardioverter-defibrillators, and biventricular pacemakers, entered into the CART-EP program during the period from 10/2007–9/2015 were considered for inclusion (N = 5753). Given the volume of CIED procedures performed and relatively low prevalence of CIED infections, an enhanced sampling approach was undertaken; details of the sampling process are described in prior published work and included in Supplementary Methods S1^14,15^.

After development of the cohort, a sample of cases underwent manual review by a trained clinician (AA, PM, WBE) to identify CIED infections that occurred within 90 days of the index procedure, applying standard cardiac device infection definitions based on clinical symptoms, microbiology results, and/or clinician diagnosis, based on recommendations from the Centers for Disease Control National Healthcare Safety Network and multi-society guidelines^16–18^. Further details on cohort development and the manual review process, including how infections were measured and defined as well as the other variables collected, are available in Supplementary Methods S1 and S2 and related references^14,15,17^.

### Diagnostic and treatment pathways and selection of potential identifying variables

#### Diagnostic variables

**Figure 2:**
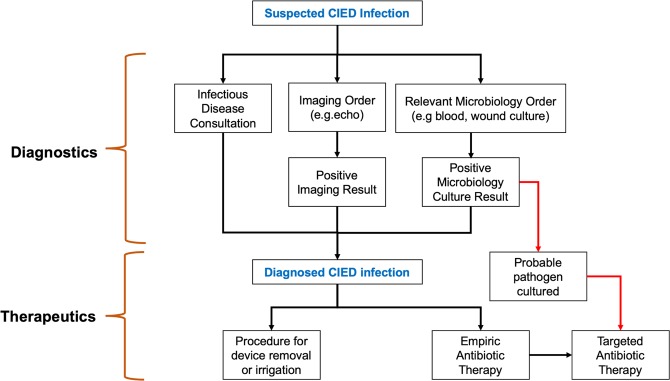
Clinical process of CIED infection cases.

CIED infections can be diagnosed and treated in several ways (Fig. 2); these interventions generate orders and results that can potentially be leveraged for retrospective infection detection^17^. Many infections are first identified due to fever, which is recorded in vital signs data as a continuous numeric variable. Microbiologic testing generates an order, which is a structured data element in the EHR, and a result, which is unstructured and not standardized. Microbiologic cultures can be obtained from the site of the device (e.g., wound cultures) or can be blood cultures, used to diagnose systemic infection, such as endocarditis. Thus, there are four potential flags associated with microbiologic testing to diagnose CIED infections: 1) a blood culture order, 2) a wound culture order, 3) wound culture result and 4) a blood culture positive result. Microbiology testing is important because it is an essential part of infection diagnosis and an essential aspect of most infection surveillance definitions. If cultures are positive, they can also provide information about the organism causing the infection and guide antibiotic treatment. Diagnosis of CIED infections may also involve imaging procedures, such as echocardiography, often transesophageal echocardiography, due to how endocarditis is defined. Mechanisms of echocardiography ordering and reporting of results are highly variable and not standardized across the VA EHR.

#### Treatment variables

Treatment of CIED infections generally involves incision and drainage of fluid collections, which may generate a structured procedure code, antimicrobials, which generate an antimicrobial order, and, in many cases, removal of the CIED with later replacement, thus generating a subsequent current procedural terminology (CPT) code. Another aspect of treatment is often clinical consultation, often with either infectious diseases or cardiology. Consultation is also recorded in various places throughout the EHR (clinical notes, orders, consult section) and is thus not a standardized data element.

### Development of the surveillance algorithm

Based on diagnostic and treatment pathways, we identified potentially relevant variables to develop the infection detection algorithm using structured data elements stored in CDW. Administrative billing codes, which are highly structured, were also considered for potential inclusion. Specifically, we used the Inpatient and Outpatient tables to obtain CPT and International Classification of Disease (ICD) 9 and 10 codes (Supplementary Methods S2), pharmacy tables for drug order data, Microbiology tables for laboratory orders, and vitals tables. These variables were classified as diagnostic of infection, treatment of infection, or billing for infection-related healthcare utilization. Unstructured variables, such as microbiology results, echocardiography completion, and consultation orders, where not considered for inclusion in the final identification model due to programming complexity and high facility-level variation in the text data; thus, inclusion of these types of variables was felt to limit generalizability. However, these manually extracted variables were evaluated as part of a sensitivity analysis to determine if their inclusion would improve algorithm performance if available and searchable in some EHRs. In the sensitivity analysis, microbiology results were further sub-classified into positive cultures with a likely infecting organism (probable pathogen) and positive cultures likely representing contamination.

### Statistical analysis

Demographic and clinical characteristics of CIED patients by infection status were compared. Chi-squared tests were used to compare categorical variables and Mann–Whitney Wilcoxon tests were used for continuous variables.

In order to build and test the infection surveillance algorithm, we randomly split the data into a training set, including approximately two-thirds of the observations, and a validation set of the remaining one-third^19^. Only the electronically-available structured variables (e.g. microbiology orders, rather than results) were considered for inclusion in the final tool, to ensure scalability and portability across EHRs. Based on the univariate association with infection status and clinical reasoning, the following were evaluated for inclusion in the flagging model: presence of a microbiology order, repeat procedure status, fever, blood, wound, antibiotic post-procedure, and a limited list of antibiotics commonly used to treat CIED infections post-procedure (Supplementary Note S3) SSI code (998.x), other CPT or ICD infection code (categorized as general infection code, material infection code or procedural infection code), and elective status for the procedure, which is routinely entered into the CART database at the time of the procedure.

Binary logistic regression was used to model the probability of an infection in the training data and chose the best combination of identifiers using the R function *bestglm*^20^, based on the Akaike information criterion (AIC), which is an estimate of relative quality of statistical models for a given set of data. We then used the GLM function with the caret package^21^ (with 3-fold cross-validation with 5 repeats, twoClassSummary and smote sampling in order to account for the rare outcome) in order to apply the model to the training data and validate on the test data. We used Youden’s method to choose the probability threshold to define positive infection status and to estimate the model performance measures^22^. To ensure model quality and robustness, we assessed calibration of the model using the givitiR package; a model is well calibrated if the predicted probabilities generated by the model accurately match the observed proportions of the response^23^. The goodness of fit of a logistic regression model can also be expressed by pseudo R-squared statistics, and we calculated Nagelkerke’s pseudo R^2^ of the logistic regressions of the training and test data using the selected predictors. This statistic is based on the log likelihood for the full model compared to the log likelihood for the baseline intercept-only model and rescaled to cover the full range of possible R^2^ values from 0 to 1^24,25^. We also explored a second modelling approach using elastic net regression and accounting for hospital-level correlation using the *glmnet* method in the caret package with a grid search to evaluate the best choice of alpha and lambda. All analyses were completed using SAS software version 9.4 (SAS Institute, Cary, NC) and R v3.5.0^26^.

## Results

The VA CART-EP database captured 5,753 CIED procedures from FY 2008–2015, representing 39 VA medical centers. 2,107 procedures among 2,068 CIED patients were manually reviewed (Table 1). The majority of patients were male (N = 2024/2068, 97.9%) and Caucasian (N = 1784/2068, 86.3%). Patients included in the cohort had a high rate of medical comorbidities; among the most prevalent were tobacco use (N = 1041, 50.3%) and diabetes (N = 977, 47.2%). Among manually reviewed cases, 97 CIED infections in 95 patients were identified within the 90-day surveillance window. Full baseline demographic details are shown in Table 1 and additional information is available in Table 1 of a related reference^15^.

**Table 1:** Patient Characteristics for Index Device Procedure by Infection Status.

Variable	Total (N = 2068)	No CIED infection* (N = 1973)	CIED Infection* (N = 95)	P-value
Training Set	1,362 (65.9%)	1,299 (65.8%)	63 (66.3%)	0.92
Validation Set	706 (34.1%)	674 (34.2%)	32 (33.7%)	
**Demographics**				
Age (Median (IQR))	71.7 (64.4–81.0)	71.9 (64.5–81.1)	68.6 (62.2–79.3)	0.06
Male Sex	2,024 (97.9%)	1,931 (97.9%)	93 (97.9%)	>0.99
Race				
White	1,784 (86.3%)	1,698 (86.1%)	86 (90.5%)	0.29
Black	248 (12.0%)	239 (12.1%)	9 (9.5%)	
Other	36 (1.7%)	36 (1.8%)	0 (0.0%)	
Hispanic	148 (7.2%)	140 (7.1%)	8 (8.4%)	0.62
**Comorbidities**				
Diabetes	977 (47.2%)	931 (47.2%)	46 (48.4%)	0.81
Tobacco Use	1,041 (50.3%)	989 (50.1%)	52 (54.7%)	0.38
Chronic Obstructive Pulmonary Disease	631 (30.5%)	595 (30.2%)	36 (37.9%)	0.11
Cerebrovascular Disease	482 (23.3%)	452 (22.9%)	30 (31.6%)	0.051
**Peripheral Arterial Disease**	**473 (22.9%)**	**438 (22.2%)**	**35 (36.8%)**	0.0009
Chronic Kidney Disease	675 (32.6%)	645 (32.7%)	30 (31.6%)	0.82
Dialysis	66 (3.2%)	62 (3.1%)	4 (4.2%)	0.54

Abbreviations: CIED = Cardiac Implantable Electronic Device.

*Determined through manual review.

Univariate analysis identified several variables that were significantly associated with true CIED infection (Table 2). Diagnostic variables included presence of a wound or blood culture order (89/97 infections vs 453/2010 uninfected controls, p < 0.0001). Wound culture orders (55/97 vs 144/2010, p < 0.0001) and blood culture orders (78/97 vs 425/2010, p < 0.0001) were also independently associated with true infections. Therapeutic flags associated with true infection included a drug order for the limited list of antibiotics after a 72-hour window post-procedure (94/97 vs 884/2010, p < 0.0001). Billing code identifiers for SSI (73/97 vs 111/2010, p < 0.0001; list of codes in Supplementary Methods S2 as well as general infection codes (34/97 vs 109/2010, p < 0.0001) were significant identifiers; material infection and CPT infections were not. A summary of antibiotic treatment regimens against identified culprit pathogens of CIED infection cases is shown in Supplementary Table S4.

**Table 2:** Procedural Characteristics for Device Procedures by Infection Status.

Variable	Total (N = 2107)	No CIED infection* (N = 2010)	CIED Infection* (N = 97)	P-value
Training Set	1,387 (65.8%)	1,323 (65.8%)	64 (66.0%)	0.97
Validation Set	720 (34.2%)	687 (34.2%)	33 (34.0%)	
***Type of device***				
Biventricular Pacemaker	36 (1.7%)	34 (1.7%)	2 (2.1%)	0.68
Biventricular Pacemaker-ICD	282 (13.4%)	267 (13.3%)	15 (15.5%)	0.54
**Permanent Pacemaker**	**1,204 (57.1%)**	**1,159 (57.7%)**	**45 (46.4%)**	0.028
ICD	600 (28.5%)	565 (28.1%)	35 (36.1%)	0.089
***Procedural variables***				
Elective procedure	1,589 (75.4%)	1,526 (75.9%)	63 (64.9%)	0.014
Index was revision procedure*	787 (37.4%)	**734 (36.5%)**	**53 (54.6%)**	0.0003
Repeat CIED procedure	91 (4.3%)	85 (4.2%)	6 (6.2%)	0.35
***Diagnostic variables***				
**Microbiology order**	542 (25.7%)	**453 (22.5%)**	**89 (91.8%)**	<0.0001
ID consult recorded*	122 (5.8%)	**78 (3.9%)**	**44 (45.4%)**	<0.0001
**Fever**	23 (1.1%)	**19 (0.9%)**	**4 (4.1%)**	0.019
Culture positive*	99 (4.7%)	**49 (2.4%)**	**50 (51.5%)**	<0.0001
**Wound culture order**	199 (9.4%)	**144 (7.2%)**	**55 (56.7%)**	<0.0001
Wound culture positive*	55 (2.6%)	**14 (0.7%)**	**41 (42.3%)**	<0.0001
**Blood culture order**	503 (23.9%)	**425 (21.1%)**	**78 (80.4%)**	<0.0001
Blood culture positive*	58 (2.8%)	**40 (2.0%)**	**18 (18.6%)**	<0.0001
Probable pathogen*	74 (3.5%)	**24 (1.2%)**	**50 (51.5%)**	<0.0001
Type of probable pathogen*				
CONS	14 (0.7%)	3 (0.1%)	11 (11.3%)	<0.0001
Enterococcus	3 (0.1%)	2 (0.1%)	1 (1.0%)	
Gram-negative Bacillus	10 (0.5%)	5 (0.2%)	5 (5.2%)	
MRSA	10 (0.5%)	1 (0.0%)	9 (9.3%)	
MSSA	12 (0.6%)	2 (0.1%)	10 (10.3%)	
Pseudomonas aeruginosa	5 (0.2%)	1 (0.0%)	4 (4.1%)	
Polymicrobial	12 (0.6%)	5 (0.2%)	7 (7.2%)	
Streptococcal sp.	6 (0.3%)	4 (0.2%)	2 (2.1%)	
Candida sp.	2 (0.1%)	1 (0.0%)	1 (1.0%)	
***Therapeutic Variables***^***a***^				
**Any antibiotic post-procedure (>72 h)**^**b**^	978 (46.4%)	**884 (44.0%)**	**94 (96.9%)**	<0.0001
**Antibiotic from limited list post-procedure (>72 h)**	880 (41.8%)	**786 (39.1%)**	**94 (96.9%)**	<0.0001
**Type of antibiotic post-procedure (>72 h)**^**c**^				
**Amoxicillin**	209 (9.9%)	**187 (9.3%)**	**22 (22.7%)**	<0.0001
**Atovaquone**	1 (0.0%)	**0 (0.0%)**	**1 (1.0%)**	0.046
**Cefazolin**	64 (3.0%)	**54 (2.7%)**	**10 (10.3%)**	<0.0001
**Cefepime**	59 (2.8%)	**50 (2.5%)**	**9 (9.3%)**	<0.0001
**Ceftriaxone**	108 (5.1%)	**93 (4.6%)**	**15 (15.5%)**	<0.0001
**Cephalexin**	184 (8.7%)	**148 (7.4%)**	**36 (37.1%)**	<0.0001
**Ciprofloxacin**	220 (10.4%)	**197 (9.8%)**	**23 (23.7%)**	<0.0001
**Clindamycin**	68 (3.2%)	**61 (3.0%)**	**7 (7.2%)**	0.023
**Doxycycline**	123 (5.8%)	**109 (5.4%)**	**14 (14.4%)**	0.0002
**Linezolid**	13 (0.6%)	**10 (0.5%)**	**3 (3.1%)**	0.019
**Minocycline**	19 (0.9%)	**14 (0.7%)**	**5 (5.2%)**	<0.0001
**Nafcillin**	7 (0.3%)	**4 (0.2%)**	**3 (3.1%)**	0.0029
**Rifampin**	8 (0.4%)	**3 (0.1%)**	**5 (5.2%)**	<0.0001
**Trimethoprim-Sulfamethoxazole**	107 (5.1%)	**85 (4.2%)**	**22 (22.7%)**	<0.0001
**Vancomycin**	274 (13.0%)	**215 (10.7%)**	**59 (60.8%)**	<0.0001
***Billing Code Variables***				
**Surgical Site Infection code**	184 (8.7%)	**111 (5.5%)**	**73 (75.3%)**	<0.0001
**General infection code**	143 (6.8%)	**109 (5.4%)**	**34 (35.1%)**	<0.0001
Material infection code	2 (0.1%)	2 (0.1%)	0 (0.0%)	>0.99
CPT infection code	1,726 (81.9%)	1,648 (82.0%)	78 (80.4%)	0.69

Abbreviations: ICD = Implantable Cardioverter-Defibrillator, ID = Infectious Disease, CPT = current procedural terminology, CONS = Coagulase-negative *Staphylococcus*, MRSA = Methicillin-resistant *Staphylococcus aureus*, MSSA = Methicillin susceptible *Staphylococcus aureus*.

*Identified from manual review.

^a^Antibiotic use only after a 72-hour window period following the index CIED procedure was applied given the frequent use of prophylactic antibiotics peri-procedure.

^b^Limited List of antibiotics in Supplementary Note S3.

^c^There was no significant difference in frequency of the following antimicrobial prescriptions between the two groups: Ampicillin, Ampicillin/sulbactam, Azithromycin, Cefpodoxime, Cefprozil, Cefuroxime, Clarithromycin, Dapsone, Demeclocycline, Dicloxacillin, Erythromycin, Gentamicin, Levofloxacin, Metronidazole, Moxifloxacin, Penicillin, Rifaximin, Tetracycline.

### Sensitivity analysis of unstructured data elements

In the CART-EP manual review data, the presence of any positive microbiologic result (50/97 vs 49/2010, p < 0.0001), as well as growth from a wound culture (41/97 vs 14/2010, p < 0.0001) or growth in a blood culture (18/97 vs 40/2010), p < 0.0001) were also potential identifiers. Identification of a probable infecting organism (e.g. *Staphylococcus* spp) was also found to be a potential identifier (50/97 vs 24/2010, p < 0.0001). Infectious diseases consultations were also positively associated with true CIED infections (44/97 vs 78/2010, p < 0.0001). However, these unstructured and variable data elements were all excluded from consideration in the final detection algorithm due to their limited potential to be included in an operational tool.

**Figure 3:**
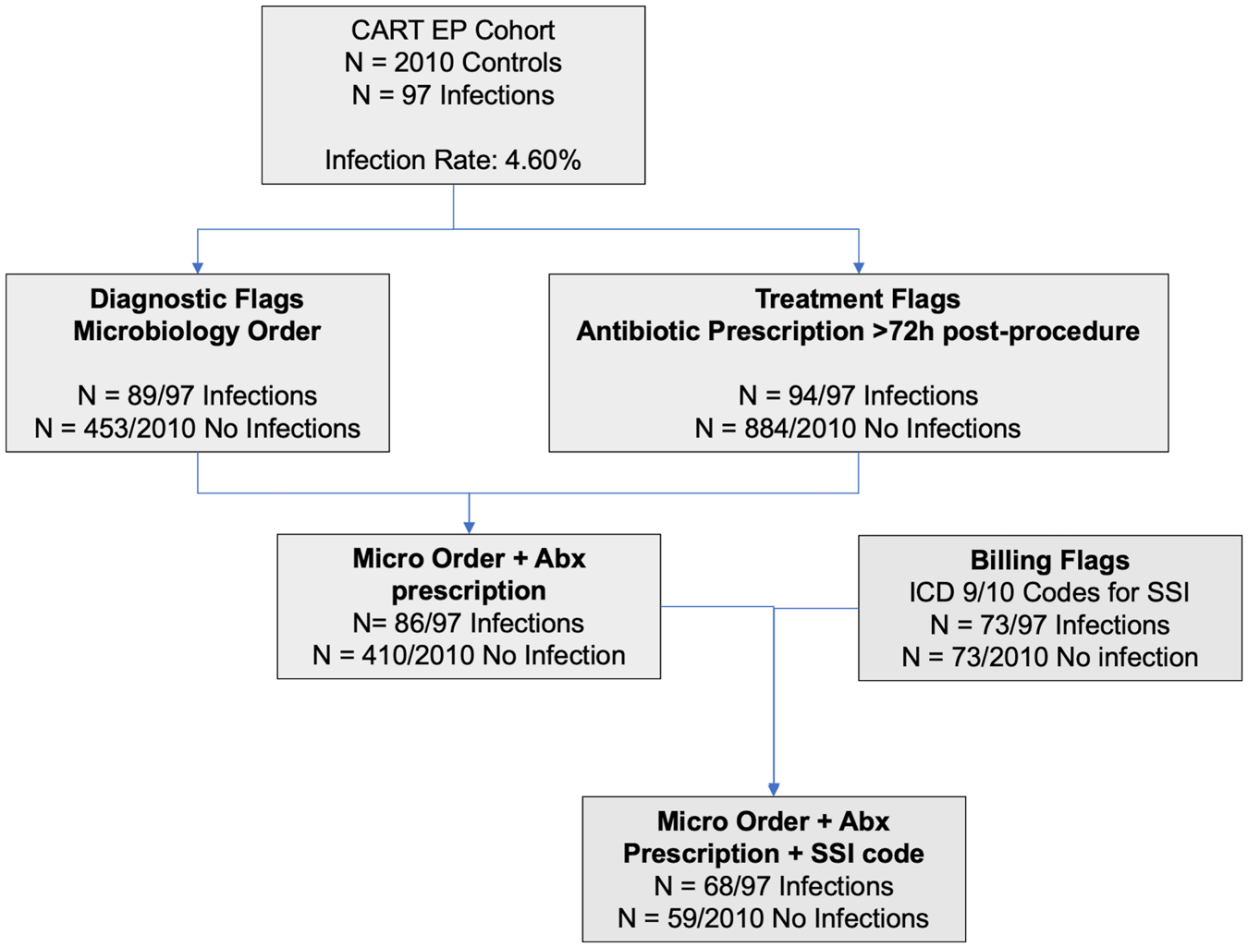
Process of using electronic identifiers to develop a detection algorithm.

The refined set of surveillance variables derived from the training set included in the surveillance algorithm is presented in Table 3 and Fig. 3. Among these, variables with the highest odds for identifying CIED infections included the presence of antibiotic order from the more limited set placed during the period>72 hours post-procedure (OR 17.76, 95% CI 4.11–76.79, p < 0.001), fever (OR 16.94, 95% CI 1.28–224.41, p = 0.032) billing code for SSI (OR 10.56, 95% CI 4.97–22.43, p < 0.001) and the presence of any microbiology order (OR 8.85, 95% CI 3.93–19.89, p < 0.001).

**Table 3:** Regression analysis of identifiers included in detection algorithm.

Identifiers	OR	95% CI	P-value
**CIED infection antibiotic post-procedure**	**17.76**	**4.11–76.79**	<0.0001
**Fever**	**16.94**	**1.28–224.41**	**0.032**
**Surgical Site Infection Code**	**10.56**	**4.97–22.43**	**<0.0001**
**Microbiology order**	**8.85**	**3.93–19.89**	**<0.0001**
**Wound culture order**	**2.95**	**1.31–6.64**	**0.0092**
Other ICD or CPT infection code	2.20	0.59–8.26	0.241721

When combined in a surveillance tool, the algorithm demonstrated high sensitivity and specificity in both the training set (84.38% and 93.58%, respectively) and the validation set (87.88% and 90.25%, respectively). The c-statistic for the algorithm in the training sample was 0.95 (95% CI, 0.92–0.98, Table 4). The negative predictive value (NPV) was 99.20% and 99.36% for training and validation sets and the positive predictive value (PPV) was 38.85% (training) and 30.21% (validation, Table 4). Compared to the rates of infection in the CART-EP data, the algorithm overestimated true infections (10.0% estimated and 4.6% observed for the training set; 13.3% estimated and 4.6% observed for the validation set). The pseudo R^2^ values for the training and validation models were 57.9% and 58.4%, respectively (Supplementary Fig. S5). We investigated using elastic net regression, including the same set of possible identifiers and accounting for correlation of observations within a hospital, but this type of model did not significantly improve either calibration or estimation.

**Table 4:** Algorithm Performance Characteristics for Training and Validation Sets.

Characteristic	Training Set	Validation Set
AUC (95% CI)	0.96 (0.94–0.98)	0.95 (0.92–0.98)
Sensitivity	84.38	87.88
Specificity	93.58	90.25
Positive-predictive Value	38.85	30.21
Negative-predictive Value	99.20	99.36

## Discussion

**Figure 4:**
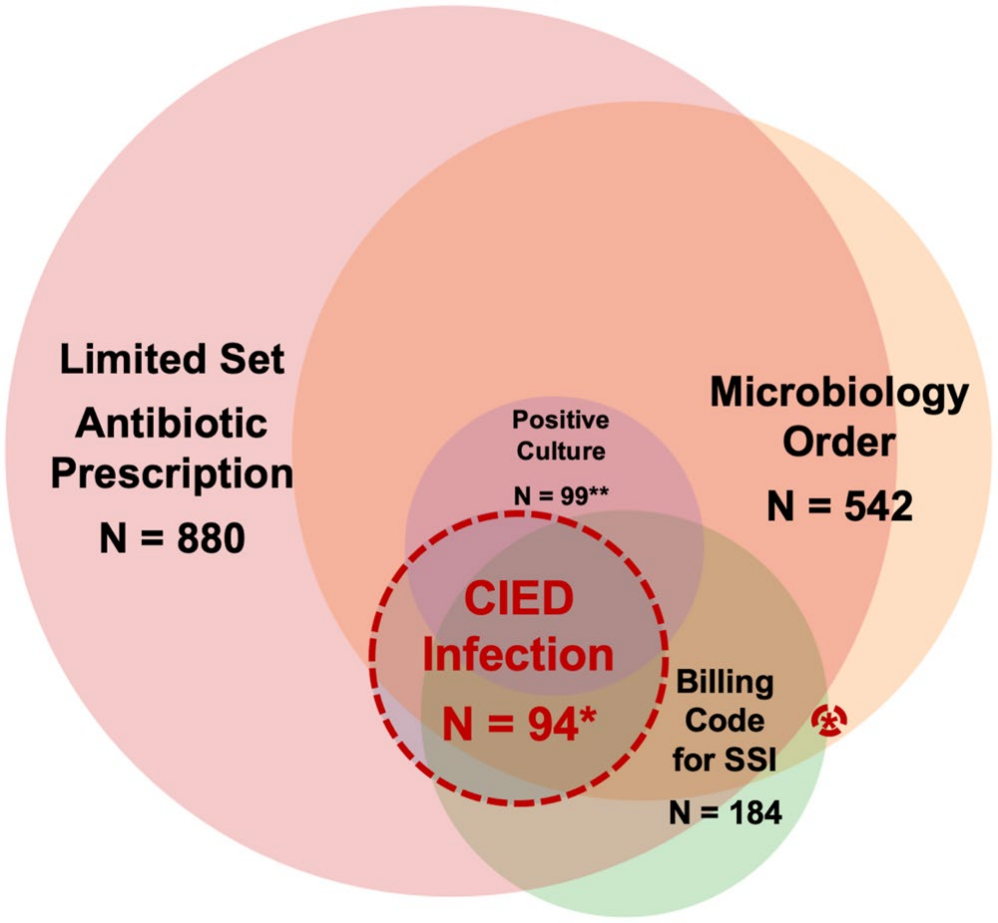
Use of electronic identifier flags in detecting CIED infection cases. Made using https://www.meta-chart.com/venn#. *3 CIED infection cases either did not have an antibiotic prescription or had antibiotics started within 72 hours of index procedure, among these three, 2 had microbiology order and positive culture and 1 had microbiology order, positive culture and billing code for SSI. **Positive growth on blood or wound culture.

Electronic, automated and semi-automated surveillance are important emerging tools in the epidemiologist’s arsenal as clinical data warehouses are increasingly available for improving bedside clinical care^27^. From a large sampling of VA CIED procedures, we found that a combination of clinically-oriented variables from CIED infection diagnostic and treatment pathways and administrative billing codes demonstrated clinically useful sensitivity and specificity for flagging true cases of CIED infection (Fig. 4). Algorithms based on structured data elements to retrospectively identify cases with a true infection can be used to expand infection surveillance to clinical care areas with limited infection prevention and surveillance coverage. Although the PPV was limited (~30%), the NPV was very high at 99%, demonstrating that the tool captured true infections well. This semi-automated flagging system could form the foundation of a surveillance program for measuring post-procedural CIED infections as part of a semi-automated process to expedite a manual review of cases most likely to have an infection; because only structured data elements are used, this tool has the potential to be easily implemented across many EHR systems.

Electronically augmented surveillance has great promise for expanding prevention to uncovered clinical areas, including procedural areas, such as the EP laboratory, and outpatient care. However, the best methodology to develop and implement these tools is an active area of research. Here, we used a methodology of first developing a list of potential identifiers based on clinical diagnostic and therapeutic pathways, and then mapped these potential identifiers to structured data elements in the EHR. A similar method of developing detection tools based on standard clinical processes could be used to expand detection to other areas with limited surveillance. In addition, EHR systems and clinical practice patterns may vary by institution, thus, a selection of the various elements identified could be customized to institutional-specific algorithms^28^. Future iterations could include advanced data extraction strategies, such as natural language processing and machine learning techniques, to further improve operating characteristics of the algorithm.

Prior work on CIED infection detection is limited. One prior single center study evaluated the utility of billing codes to measure rates of CIED infections; our data from a large, multicenter national sample suggests that this strategy may have high specificity but low sensitivity. In our study, 27.6% of true-positive cases in our analysis would not have been flagged using a system reliant on billing codes alone^29^. Our finding about the limited indicative utility of billing codes alone is similar to work in cardiac surgery, which similarly found that coding data had limited value for measuring post-surgical infections (PPV < 26%)^30–32^. The challenge of the limited sensitivity of coding data is compounded by recent data which suggests that ICD-10 codes may even lower predictive probabilities than ICD-9 codes^33^.

We expanded upon prior research into semi-automated electronic surveillance tools by including structured variables available in commonly used EHRs that could be leveraged for CIED identification. In combining these variables, we developed and validated an electronic surveillance tool with excellent specificity and sensitivity, high NPV, and reasonable PPV, increasing the rate of case identification from 4–5% to 30–40%. The high NPV of the tool highlights the importance of the absence of clinical identifiers in ruling out cases that do not have an infection; this NPV can be leveraged to greatly streamline a manual review process.

Prior work demonstrates that a major barrier to implementing surveillance in outpatient and procedural areas is limited time and resources^34,35^; a semi-automated system that is easy to program and dramatically reduces chart review has the potential to bypass some of these implementation challenges to facilitate expansion of surveillance. The substantial improvement in PPV markedly enhances the yield of a manual review process and reduces the time and resources necessary for infection detection. In addition, although the tool identified flagged some negative cases, it was calibrated to optimize case ascertainment and to triage and minimize the burden of manual review, not to replace it entirely. A combined automated and manual approach to infection detection forms a powerful tool for generating accurate data not only on infection rates but also on quality and process metrics. Identifying patient safety metrics that can be acted upon to promote improvements in infection prevention strategies is an important aspect of the response for averting additional procedure-related infections. Critical changes to patient safety and process could not be identified or implemented without the information generated during a detailed manual review and root cause analysis.

When considering implementation and adaption to other settings of care, it is useful to consider how each type of structured data element impacted the probability an adverse event occurred. For example, use of antimicrobial prescriptions post-procedure was highly sensitive but not specific. This lack of specificity is driven by the breadth of CIED infection pathogens and the diverse set of antimicrobials that can be selected to treat them^15^. Using a limited set of antibiotics—for example, training the algorithm to detect only antimicrobials directed toward common bacterial pathogens— might improve specificity but at the cost of potentially not detecting CIED infections caused by unusual, and potentially more severe, pathogens. Another approach to enhance the PPV of the tool might be to use combinations of identifiers such as drug-bug matches (i.e. specific antibiotic prescriptions that correspond to particular microbiology results) as a discrete variable. However, this would layer in additional complexity to programming and simultaneously reduce sensitivity. In our sample, approximately half of infections did not have positive blood or wound microbiology, thus, the process of requiring a microbiology result, let alone layering results with interaction terms, would miss more than half of the cases. In addition, our focus was on the use of structured data flags due to excellent NPV of these variables, and to ensure generalizability to a wide variety of EHRs. However, the use of typically unstructured data—such as information contained in clinical notes—may be an approach to improve PPV in future iterations.

The limitations of this study were largely due the dataset used to create and validate the algorithm and difficulty in operationalizing clinical variables into electronic identifiers. We relied on CART-EP data to develop and validate the algorithm, and the selection process for these CIED procedures may limit the generalizability of our findings to other VA CIED procedures and procedures performed outside of the closed VA healthcare system. Our reliance on data available in VA records could mean that CIED infection occurring outside the VA system may not have been captured. However, we manually reviewed all scanned in outside hospital records and these were included as cases in the study; if they did not have clinical variables input into the VA EHR, these would have been included as “false negatives” in the development and assessment of the tool and reflected in the operating characteristics of the model. Further, prior studies demonstrate that the majority of patients return to the closed VA healthcare system for subsequent procedural care.

Regarding operational challenges, we found that wound culture results had excellent promise for infection detection, however, due the nature of the variable as free-text and not standardized, it is not easily measured using an automated system across multiple sites with highly variable documentation practices.

Finally, healthcare systems and practices are not static, but rather constantly evolving. Thus, automated infection surveillance algorithms that leverage clinical pathways, including this CIED infection flagging tool, will require ongoing adaption and updates as novel therapeutics are introduced and diagnostic strategies evolve. Thus, detection algorithms must move toward a learning health system model, with constant modifications and adaptations to maintain their estimative utility^36^.

## Conclusions

Existing surveillance structures for CIED infections are absent in many institutions, partially due to limited resources for detection and monitoring^35^. This study demonstrates that electronic surveillance tools based on structured data elements can be developed with high sensitivity and specificity and have the potential to expand measurement with audit and feedback to clinical areas that have not been served by traditional surveillance programs. Future improvements could include strategies to enhance detection of variables primarily captured in clinical notes.

## Supplementary information

Supplementary Information.
