# Supplementary material for: Development and Validation of a Semi-Automated Surveillance Algorithm for Cardiac Device Infections: Insights from the VA CART program
Source: Sci Rep. 2020 Mar 24;10:5276. doi: 10.1038/s41598-020-62083-y (PMC7093485; doi:10.1038/s41598-020-62083-y)

Title: Development and Validation of a Semi-Automated Surveillance Algorithm for Cardiac Device Infections: Insights from the VA CART program

Archana Asundi^1^, Maggie Stanislawski^2,3,4^, Payal Mehta^5^, Hillary J. Mull^6,7^, Marin L. Schweizer^8^, Anna E. Barón^9^, P. Michael Ho^2,4,10^, Kalpana Gupta^5,6,11^, Westyn Branch-Elliman*^5,6,12^

^1^Division of Infectious Diseases, Boston Medical Center, Boston, Massachusetts, USA

^2^Seattle-Denver Center of Innovation for Veteran-Centered and Value-Driven Care, Seattle, Washington and Aurora, Colorado, USA

^3^Division of Biomedical Informatics and Personalized Medicine, University of Colorado School of Medicine, Aurora, Colorado, USA

^4^ Cardiology Section, Rocky Mountain Regional VA Medical Center, Aurora, Colorado

^5^Department of Medicine, Division of Infectious Diseases, Boston VA Healthcare System, West Roxbury, Massachusetts, USA

^6^Center for Healthcare Organization and Implementation Research (CHOIR), Boston VA Healthcare System, Boston, Massachusetts, USA

^7^Department of Surgery, Boston University School of Medicine, Boston, Massachusetts, USA

^8^Center for Access and Delivery Research and Evaluation, Iowa City VA Health Care System, Iowa City, Iowa

^9^Department of Biostatistics & Informatics, Colorado School of Public Health, University of Colorado Anschutz Medical Campus, Aurora, Colorado, USA

^10^Department of Medicine, Division of Cardiology, University of Colorado School of Medicine, Aurora, Colorado, USA

^11^Boston University School of Medicine, Boston, Massachusetts, USA

^12^Harvard Medical School, Boston, Massachusetts, US

Supplementary Methods S1: Manual Review Process

- Given low expected incidence rate of CIED infections (estimated 1-3%) cases with higher CIED infection probability were over-sampled to enhance case ascertainment.
- All procedures with a potentially relevant ICD9/10 code and/or a blood culture order and/or wound culture order within 90 days of the index device procedure underwent manual review by a trained infectious diseases clinician (AA, WBE) applying standard definitions for CIED infection.
- Cases in the enriched sample were then matched 1:3 with cases without potentially relevant ICD9/10 codes and without potentially relevant microbiology orders.
- Unenriched sample then stratified by facility to the high-probability sample
- Then, a random number generator was used to select cases for manual review.
- If a facility lacked sufficient CART-EP-entered procedures to complete the matching process, then other low-probability procedures were selected randomly from the cohort so that three unenriched procedures were matched for every one procedure in the enriched sample.
- Cases without documented cardiac device procedures and/or without clinical notes entered into the VA EHR were excluded.

Supplementary Methods S2: List of ICD-9 and -10 Codes evaluated as possible identifiers

ICD-9 Codes: 998, 996.61, E848.1; E878.8; E878.9, T81.9, 996.70, 996.72, 780.61, 780.62, 041.9, 136.9, 995.91, 994.92, 031.12, 041.12, 0.38.11, 0.41.11, 038.10, 038.19, 041.19

ICD-10 Codes: T82.7XX, T81.4XX, R50.82, B99.9, A41.9, T81.9, A49.02, B95.6, T82.6, T83.5, T83.6, T84.5, T84.7, T85

Supplementary Note S3: Antibiotics commonly used to treated culprit pathogens of CIED infections

Amoxicillin, Ampicillin/Sulbactam, Cefazolin, Cefepime, Ceftriaxone, Cephalexin, Ciprofloxacin, Clindamycin, Daptomycin, Dicloxacillin, Doxycycline, Levofloxacin, Linezolid, Minocycline, Moxifloxacin, Nafcillin, Penicillin, Rifampin, Tetracycline, Trimethoprim, Vancomycin

Supplementary Table S4: Antibiotic-Pathogen Matrix

|  | Cephalexin | Cefazolin | Penicillin | Nafcillin | Daptomycin | Vancomycin | Ertapenem | Minocycline | Amoxicillin-Clavulanate | Clindamycin | Doxycycline | Trimethoprim/Sulfamethoxazole | Linezolid | Ciprofloxacin | Moxifloxacin | Ceftriaxone | Cefepime | Unknown Antibiotic | Combination Antibiotic Regimen | No Antibiotic Treatment |
| --- | --- | --- | --- | --- | --- | --- | --- | --- | --- | --- | --- | --- | --- | --- | --- | --- | --- | --- | --- | --- |
| MSSA | 3 | 2 |  | 1 | 1 |  |  |  |  |  |  |  |  |  |  |  |  | 3 | 1 |  |
| MRSA |  |  |  |  |  | 6* |  | 1 |  |  |  |  |  |  |  |  |  | 2 | 1 |  |
| CONS |  |  |  |  |  | 2 |  |  | 1 | 1 | 1 | 1 | 1 |  |  |  |  | 4 |  | 1 |
| Other GP |  |  | 1 |  |  |  |  |  |  |  |  |  |  |  |  |  |  | 3 |  |  |
| PSA |  |  |  |  |  |  |  |  |  |  |  |  |  | 1 |  |  | 1 | 1 | 1 |  |
| Other GNB |  |  |  |  |  |  |  |  | 1 |  |  | 1 |  | 2 |  | 2 |  |  | 1 |  |
| Polymicrobial |  |  |  |  |  | 1 | 1 |  |  |  |  |  |  |  |  |  |  | 4 |  |  |
| No Growth | 5 |  |  |  |  | 3 |  |  | 3 | 1 | 1 | 1 |  | 1 |  |  |  | 9 | 6 | 1 |
| Not Available | 2 |  |  |  |  | 2 |  |  |  |  | 2 | 1 |  | 1 | 1 |  |  | 1 | 2 |  |

Abbreviations: MSSA = Methicillin-sensitive Staphylococcus aureus, MRSA = Methicillin-resistant Staphylococcus aureus, CONS = Coagulase-negative Staphylococcus, GP = Gram positive, PSA = Pseudomonas aeruginosa, GNB = Gram-negative bacilli

*One of these cases had antibiotics initiated within the 72-hour window applied for the detection algorithm.

Supplementary Figure S5: ROC Curves of GLM model for (A) Training and (B) Validation sets


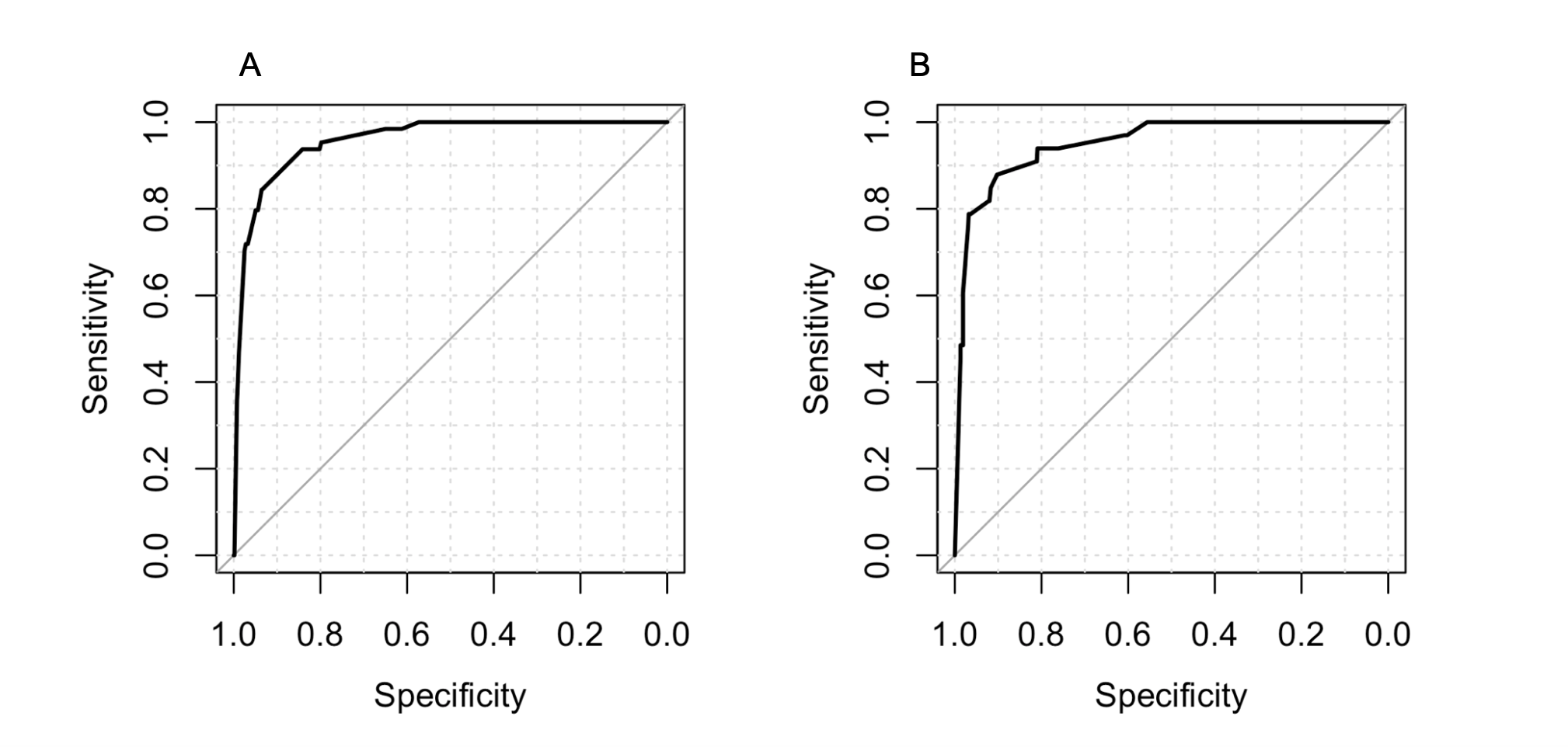

Supplement: Supplementary file 1 — Supplementary Information. [file 41598_2020_62083_MOESM1_ESM.docx]
